# Identification of microRNA precursors based on random forest with network-level representation method of stem-loop structure

**DOI:** 10.1186/1471-2105-12-165

**Published:** 2011-05-17

**Authors:** Jiamin Xiao, Xiaojing Tang, Yizhou Li, Zheng Fang, Daichuan Ma, Yangzhige He, Menglong Li

**Affiliations:** 1 College of Chemistry and State Key Laboratory of Biotherapy, Sichuan University, Chengdu 610064, P.R. China

## Abstract

**Background:**

MicroRNAs (miRNAs) play a key role in regulating various biological processes such as participating in the post-transcriptional pathway and affecting the stability and/or the translation of mRNA. Current methods have extracted feature information at different levels, among which the characteristic stem-loop structure makes the greatest contribution to the prediction of putative miRNA precursor (pre-miRNA). We find that none of these features alone is capable of identifying new pre-miRNA accurately.

**Results:**

In the present work, a pre-miRNA stem-loop secondary structure is translated to a network, which provides a novel perspective for its structural analysis. Network parameters are used to construct prediction model, achieving an area under the receiver operating curves (AUC) value of 0.956. Moreover, by repeating the same method on two independent datasets, accuracies of 0.976 and 0.913 are achieved, respectively.

**Conclusions:**

Network parameters effectively characterize pre-miRNA secondary structure, which improves our prediction model in both prediction ability and computation efficiency. Additionally, as a complement to feature extraction methods in previous studies, these multifaceted features can reflect natural properties of miRNAs and be used for comprehensive and systematic analysis on miRNA.

## Background

MicroRNAs (miRNAs) are short non-coding RNA molecules of ~22 nucleotides (nt) that can affect stability and/or translation of mRNAs. In mammals, the primary transcript (pri-miRNA) is processed into a precursor (pre-miRNA) of ~70 nt with a characteristic stem-loop structure by the enzyme, *Drosha*, and then the pre-miRNA is transported from nucleus to cytoplasm by *exportin-5*. The Dicer nuclease cuts out the mature miRNA from one strand of the pre-miRNA, and loads it into RNA-induced silencing complex (RISC) [1-3]. Finally, the cleavage or translational repression is induced, depending on the degree of base pairing between RISC-miRNA and target mRNA [4,5].

The first miRNA, lin-4, was discovered in the lab of Victor Ambros in 1993 [6], which was complementary to the 3'UTR (3' untranslated region) of the mRNA transcribed from the lin-14 gene. Seven years later, the second miRNA let-7 was found in Gary Ruvkun's lab [7]. Subsequently, miRNAs have become a hot spot and a large number of miRNAs have been identified in various species across time [8-12]. There are 10883 mature miRNA products, according to the release 14.0 of miRBase [13]. MiRNAs are integral components in many biological processes including development, differentiation, apoptosis, etc. Moreover, unexpected novel functions have been discovered recently. However, the experimental techniques are difficult to systematically detect miRNA molecules under the assumption that highly diverse functions and activities are involved in biological processes.

At present, computational methods, including comparative and non-comparative methods, prove good at identifying miRNA precursor from both pseudo miRNA and other ncRNA (non-coding RNA), which are also available for human pre-miRNA recognition. Xue *et al*. (2005) presented a classifier (*triplet-SVM*) based on support vector machine to classify human pre-miRNA from pseudo hairpin with structure-sequence triplet features. *MiPred *extended the *triplet-SVM*, using the random forest algorithm based on hybrid features to improve the classification results [14,15]. To the contrary, *miPred *and *microPred *regarded some other ncRNAs (such as tRNAs and rRNAs) as a negative training/testing dataset for the reason that pseudo hairpin structures can be found in the complete secondary structures of other types of ncRNAs and their motifs. Therefore, a proper approach for novel human pre-miRNA recognition should distinguish real pre-miRNA hairpins effectively, from both genome pseudo hairpins and other ncRNAs [16-18].

Almost all pre-miRNAs have characteristic stem-loop hairpin structures, which are thought to provide insight into the biological function [19]. During the biogenesis of a mature miRNA, hairpin structure acts as a structure motif for Exportin-5 in the nuclear-cytoplasm transportation and a substrate for Dicer [20-22], and it is also of great importance in the specific nucleotide base-paring and stacking interactions. In the RNA folding, the adopted shapes or folds can be highly complex while capable of carrying out a variety of molecular functions, such as binding metabolites and proteins with high specificity [6,23-27]. Genomic regions are also binding targets for RNAs allowing for their hybridization with nucleotide sequences [28-30]. As for researches into microRNA function, the identification of miRNA targets using computational methods has developed in an increasing number. Recent improvements in this field have been reported in Li J *et al *[31], *Target-align *[32], *MiRonTop *[33], etc.

**Figure 1:**
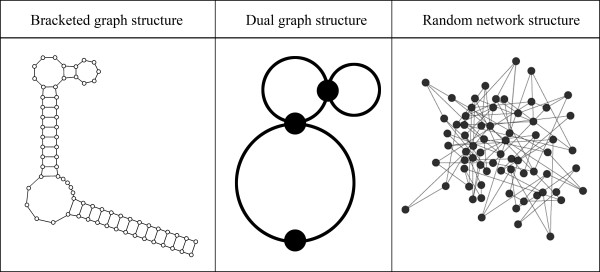
**Three representations of RNA secondary structure for human precursor miRNA hsa-mir-33a**.

Recent studies showed various ways to represent RNA structure with graphs (Figure 1), such as bracketed, tree, dual graph, etc. These representations specify the connectivity between RNA secondary structural elements, such as loops, bulges, stems and junctions [34,35]. They facilitate the detection of numerous detailed facets of each pre-miRNA element and their combined patterns in creating pre-miRNA secondary structure. Thus, a parameter can be defined on the level of network constituents (i.e. nodes and edges) or the network itself. In this work, we describe a pre-miRNA secondary structure as a two-dimensional network (graph), and then several network parameters are defined and analyzed. Based on these parameters, a random forest (RF) approach is used to construct prediction model for pre-miRNA. This classifier is trained on animal pre-miRNA sequences with <90% similarity and achieves high accuracies across independent datasets.

## Results and Discussion

### Prediction performance of RF classifier

#### Training/testing model

Embedded in the procedure of estimating classifier performance, the parameter optimization is done by grid search. During the process of the grid search, two parameters, *ntree *(number of trees to grow) and *mtry *(number of variables randomly sampled as candidates at each split) are optimized based on 10-fold cross-validation. The original value is *ntree *= {500, 2000, 500} and *mtry *= {0, mdim, 1} (the first number indicates the initial value, the second indicates the final value, the third is the step size, and the mdim is the number of features). The best performed parameters (*ntree *= 1500, *mtry *= 8) are selected to construct random forest prediction models. A similar method of parameter optimization was also successful in predicting miRNA targets [36].

A RF model is constructed with training dataset and tested by testing dataset. Our dataset contains 3928 positive samples (animal pre-miRNAs) and 8897 negative samples (pseudo hairpins and other ncRNAs). 3000 samples from each class are randomly selected for training, and the rest are for testing. This procedure is repeated 100 times and the true positive rates (sensitivity) and the true negative rates (specificity) are averaged to determine the performance. Our method achieves sensitivity of 0.873 and specificity of 0.911. Comparing with previous report, our approach is well performed, as redundant sequences are filtered out with a threshold of 90% identity while others only get rid of duplicate sequences. The stricter data preprocessing reduces the bias of prediction results yielded by redundant data.

**Figure 2:**
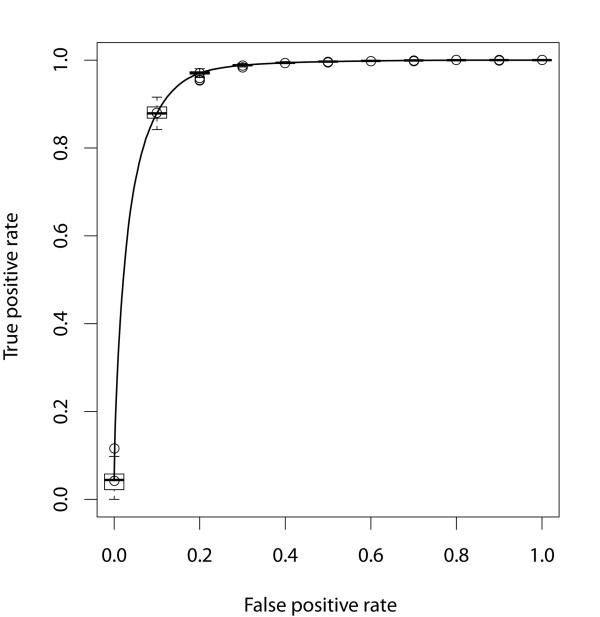
**ROC curves estimate the random resampling models**. The ROC curves are overlaid by the vertical average curve and box plots showing the vertical spread around the average.

ROC curves for testing datasets represent the distribution of 100 times experiments with box plot, in which the middle bar is the median, the outer edges are the 10 and 90 percentiles, and the edges of the boxes are the 25 and 75 percentiles. Outliers are showed as circles. An average AUC value of 0.956 is obtained with all network parameters (Figure 2). The result further suggests that network represented stem-loop secondary structure can be used to construct model for effectively predicting novel pre-miRNA.

#### Performance of independent dataset

In order to evaluate the practical prediction ability of the final prediction model, two independent datasets are used, which contains 1646 known plants and 196 virus pre-miRNA sequences, respectively. Table 1 shows the results on the independent datasets. Sequences with similarity greater than 90% are excluded from original dataset. Our model achieves high accuracies of 0.976 and 0.913, respectively. A total accuracy of 0.970 indicates that our method is reliable and robust. Network parameters can be used to identify pre-miRNA sequences with high performance. In contrast, most existing methods only work on the pre-miRNAs with no multiple loops, and do not filter out the high similarity sequences. The pre-miRNAs sharing high sequence similarities induces biased evaluation of the prediction model in this manner.

**Table 1:** Comparison with existing methods

Methods	Complete dataset		Training dataset		Testing dataset		Results for testing dataset		Results for Independent dataset		
	
	Pos	Neg	Pos	Neg	Pos	Neg	SE	SP	Plant (Acc)	Virus (Acc)	Total (Acc)
Triplet-SVM	193	1168	163	168	30	1000	0.933	0.881	0.882	0.843	0.877^a^
microPred	691	9248	SMOTE Outer-5-fold-CV				0.900	0.973	0.841	0.939	0.853^b^
Our method	3928	8897	3000	3000	928	5897	0.873	0.911	0.976	0.913	0.970

*Triplet-SVM *is a SVM-based method with triplet elements that represent information of pre-miRNA stem-loop structure. There is an extension called *MiPred*.

*microPred *combined the new RNAfold-related, Mfold-related, and pair-related features with 29 'global and intrinsic' features introduced in the *miPred *approach.

^a ^178 virus and 1232 plant sequences were used, as samples with multiple loops were filtered out by *Triplet-SVM*.

^b ^196 virus and 1389 (the length less than 300) plant sequences were submitted to *microPred *web server.

### Contribution of individual parameter

In the present work, two different strategies are adopted to measure the contribution of individual parameter to the prediction of pre-miRNA. Because predicting the response with "black-box" model alone cannot fully satisfy the requirements in the current classification tasks.

**Figure 3:**
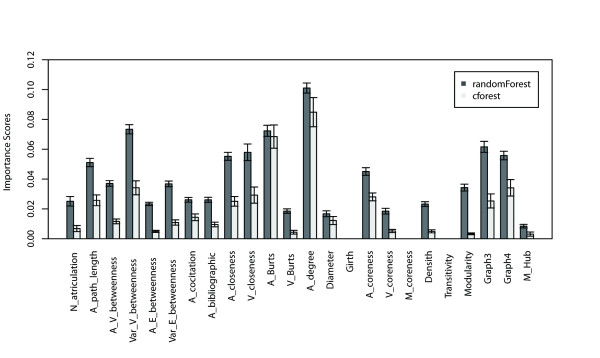
**The bar charts of individual parameter contribution**. The contribution of individual parameter is determined by calculating the importance score, with larger scores indicating more relevant properties. The comparison between two strategies is represented by different greyscales, the bar height is the score of individual feature, and the confidence interval is calculated for each parameter. E: Edge; V: Vertex; N: Number; A: Average; Var: Variance; M: Mean.

RF is a classification method that also provides feature importance measures, with which significant features would be distinguished and interactions among features would also be reduced as well. Permutation importance and conditional variable importance are adopted as criteria for measuring the contribution of individual parameter in pre-miRNA prediction. This process is repeated 100 times with random resampling of constructed models, and the scores are averaged. The contribution of each network parameter is measured and showed in Figure 3.

**Figure 4:**
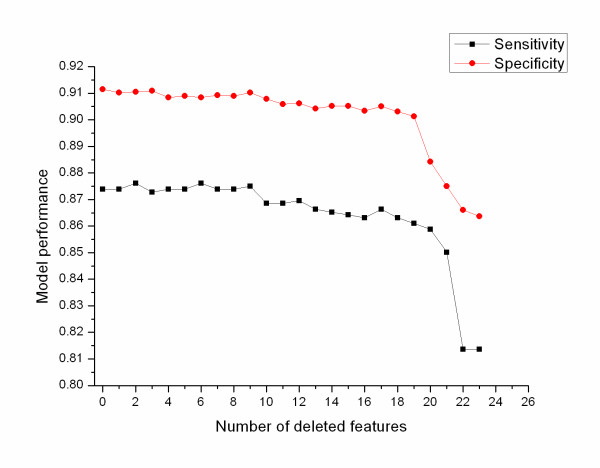
**Results for deleting feature one by one**. Models are constructed on remainder variables after deleting the feature of the lowest score each time. This process is repeated 23 times, till only one feature is left. Sensitivity and specificity are used to measure model performance.

The average degree exhibits the greatest contribution with an average score of 0.1010 followed by the variance of betweenness (0.0734), the average Burt's constraint (0.0723), and three graph motifs (0.0616). These parameters significantly contribute to the performance of the model and are consistent with the results from permutation and conditional variable importance strategies. However, the latter based on conditional inference trees seems to produce less noise than a permutation importance strategy. In addition, this analysis suggests that girth, coreness and transitivity have a limited or no contribution to the prediction. Subsequently, we rank the features by average score of each parameter, and delete one feature of the lowest score each time and construct models with features remained. This procedure is repeated 23 times, till only one feature is left. The average prediction results for each model are showed in Figure 4. The complete parameter set is tested in the classifier that achieves sensitivity of 0.873 and specificity of 0.911. Elimination of lower scored parameters does not lead to significant change of the model performance. When the top 4 parameters are remained, the sensitivity is 0.859 and specificity is 0.884, decreasing by 0.014 and 0.027 respectively, comparing to the results from the total feature sets. These results further confirm the above experiment, and the top 4 parameters are of great contribution to pre-miRNA prediction.

### Comparing the practical prediction ability with previous methods

Several pre-miRNA prediction tools were released and each had its own merits. However, these tools suffered from imbalance problem, that is, the negative dataset was much larger than the positive dataset. The *triplet-SVM*, *MiPred *and *miPred *all randomly chose more balanced positive and negative dataset from the complete dataset as the training data. Meanwhile, the remaining positives and other randomly chosen negatives were as the testing samples. Instead, *microPred *used SMOTE (Synthetic Minority Over-sampling Technique) to address this imbalance problem. However, SMOTE and random over-sampling methods augment the minority class through all the samples or a random subset of the minority class. Over-sampling makes exact copies of the minority class, which tends to result in over-fitting of the model. Thus, these methods increase the size of the training set to build a classifier, which likely yields overestimated prediction ability. In addition, existing methods haven't considered redundant owing to high similarity sequence, which results in biased evaluation of the prediction performance. Here, 3000 samples are randomly selected from 3928 non-redundant animal sequences with less than 90% similarity and 8897 negative samples (8487 pseudo pre-miRNAs and 410 ncRNAs), respectively. The remainder samples are used for testing model. Finally, 1646 plants and 196 virus sequences <90% similarity are used to evaluate the practical prediction ability of the final prediction model and compare with that of the previous tools.

We perform a comparison on the independent dataset, and the result is listed in Table 1. Triplet elements were first proposed in *triplet-SVM*, which combined the local contiguous sequence and structure information of the stem-loop secondary structure of pre-miRNA [14]. This feature representation could be effectually applied in pre-miRNA identification, which was further proved and improved by recent study [15]. The *microPred *presented 48 multifaceted features, including 29 conventional features originally used in the *miPred *approach and 19 newly introduced RNAfold-related, Mfold-related, and pair-related features [16,17]. In our approach, a novel representation of pre-miRNA structure is proposed by translating characteristic stem-loop structure into network and generating 24 network features for random forest classification. The independent dataset test indicates that all three methods perform well, and our model performs best.

Besides, we have also implemented our method on the same positive and negative datasets previously used. We train our model with the same training data as that of the *triplet-SVM*, and test model performance with the same testing data used in *triplet-SVM*. As a result, all the 30 human pre-miRNAs are correctly recognized, while 895 out of 1000 pseudo-miRNAs are defected as negative by our method. Comparing with *triplet-SVM*, this method significantly improves the performance of prediction. Moreover, we try our approach on positive and negative datasets originally used in *microPred*, yielding sensitivity of 0.889 and specificity of 0.901. The *microPred *was time-consuming, as a large number of random sequences were generated for calculating statistical thermodynamic features. Our method not only yields high accuracy, but also greatly reduces the computation time. This result demonstrates that our method is robust and effective. Network parameters can be considered as a complement to feature extraction of previous work, using in comprehensive and systematic feature analysis for pre-miRNA prediction.

## Conclusions

MicroRNA investigation not only sheds new light on RNA function, but also reveals the mechanism involved in cell function and regulation. Current methods use sequence, triplet structure-sequence, and thermodynamic properties to construct prediction model of pre-miRNA. In the present study, we design a novel representation of pre-miRNA secondary structures for modelling pre-miRNA classifier. The graph theory is applied in analyzing RNA structure recently, and some of the relevant biological relations can be explained. For instance, the node betweenness is thought to measure the number of base pairs existed in the structure and the compactness of structure to a small extent. Likewise, the articulation point is regarded as a nucleotide in a dangling end or a bridge between two separable secondary structures [37]. However, further researches of biological interpretations for graph properties are in demand. As more and more new methods have been proposed, we are highly assured that understanding microRNA and complex biological processes they influence could unlock the secrets of their function.

## Methods

### Random forest

Random forest (RF) consists of many unpruned decision trees and the outputs are decided by the predictions of all the individual trees for both classification and regression. All the trees vote to determine the prediction result and an OOB estimate of error rate is implemented. As a classifier, random forest is constructed of *ntree *trees grew from different bootstrap samples using original data, and splits each node by the best split among randomly sampled *mtry *predictors at that node. It combines bootstrap aggregating (bagging) algorithm and the random feature selection to construct a collection of decision trees with controlled variation. Bagging is used to improve Mach Learn of classification and regression models in terms of stability and accuracy. It also reduces the variance and helps to avoid overfitting [38]. However, when the measure is based on the predictor's performance in the training set, there is no possibility of knowing whether the predictor is over-fitted to the training set. Instead, cross-validation should be used to test the performance of predictor. The RF algorithm has been successfully applied in situations where complicated interactions are among many features. Based on a tree structure, it has advantages of interpretable classification rules and additional information to measure the importance of features. Feature extraction is a difficult issue owing to the complexity of interactions between different features. However, only predicting the model response cannot be achieved for many applications. The random forest algorithm for classification, regression and variable importance measurements is available in the randomForest and the party R packages.

### Training and Testing dataset

A total of 8531 animal pre-miRNA sequences are collected from miRBase14 [13]. The redundant sequences are filtered out with a threshold of 90% sequence identity, retaining 3928 non-redundant sequences. Then the remainders are folded into stem-loop secondary structures by UNAfold. We consider all these 3928 non-redundant pre-miRNA sequences as our positive dataset whether multi-branched loops exist or not.

The 8494 human pseudo pre-miRNAs have been previously used in several works [14-17]. This dataset is downloaded from Xue *et al*.'s work collected from protein coding region. More likely pseudo hairpin sequences do not contain any annotated or un-annotated pre-miRNA sequences. Additionally, other ncRNA samples are considered as negative dataset from Batuwita *et al*.'s work. These ncRNAs have pseudo hairpins, which resemble pre-miRNA in structure much more. This dataset was originally formed by the automatic prediction methods with the predicted pseudo-genes removed manually and carefully [39,40]. Taking other ncRNA sequences into consideration enriches the negative dataset by providing additional information representing their hairpin motifs. Similarly, redundant sequences are filtered out with a threshold of 90% sequence identity, and the remainder sequences are folded into stem-loop structures. Finally, we obtain 8487 pseudo pre-miRNAs and 410 ncRNAs in the negative dataset.

### Independent testing dataset and evaluation index

Two cross-species datasets, plant and virus pre-miRNAs, are downloaded from miRBase as independent datasets to test our classifier. After processing the original data, 1646 plant and 196 virus pre-miRNAs are obtained to evaluate the practical prediction ability of the classifier. The whole data preprocessing is parsed with *Perl*.

Finally, sensitivity (SE), specificity (SP), and the total prediction accuracy (ACC) are used to measure the performance of this method, which are defined as follows:

(TP, TN, FP and FN represent true positive, true negative, false positive, and false negative, respectively). The sensitivity for positive prediction, the specificity for negative prediction, the accuracy for total prediction, and ROC plots of the true positive rate versus the false positive rate for varying decision cut-offs, are used to measure the model performance.

### Extraction of network parameters

Network elements, including nodes and edges, can be defined by the network itself or a parameter which may relate to limited or full knowledge of the network. Based on these criteria, Child *et al *classified the network parameters into three types: local, local-global and global (using limited or full knowledge of the network and referring to a network element or network itself) [37]. Thus, the network represented pre-miRNA structure offers a means to capture both local-global and global structural properties that can be used as a novel method in identification of miRNA. To our knowledge, the characteristics of diverse biological processes or the ensemble can be reflected by modelling the network.

Here, 24 network parameters are adopted to describe stem-loop structure of pre-miRNA based on previous work and experimental criteria [41,42] although a number of network parameters are available. Individual parameter definition is listed in Table 2. UNAfold tool is used to predict pre-miRNA secondary structure represented of bracketed graph, which converts all nucleotides to nodes and all bonds between nucleotides (both ester and hydrogen) to edges. Moreover, the necessary summary statistics (mean and variance) are performed to extend the present algorithm in calculating some parameters based on individual node or edge. For example, for edge betweenness, the mean and the variance over all edges in the graph are calculated. All network parameters are calculated with the igraph R package [43].

**Table 2:** Definition of network parameter

Parameter	Description
Hub score	Kleinberg's hub.
Path length	The length of a path.
Shortest path	The shortest path between two vertices.
Constraint	Calculates Burt's constraint for each vertex.
Degree	The number of edges connected to a vertex.
Grith	The length of the shortest circle in the graph.
Modularity	Modularity of a community structure of a graph.
Graph motifs	The small subgraphs with a well-defined structure.
Articulation point	A vertex that, if removed, will disconnect the graph.
Node betweenness	The number of shortest paths that pass through a vertex.
Edge betweenness	The number of shortest paths that pass through an edge.
Diameter	The diameter of a graph is the length of the longest geodesic.
Cocitation coupling	Two vertices are cocited if there is another vertex citing both of them.
Transitivity	Measures the probability that the adjacent vertices of a vertex are connected.
Bibliographic coupling	The bibliographic coupling of two vertices is the number of other vertices they both cite.
Closeness centrality	Measures how many steps are required to access every other vertex from a given vertex.
Coreness	The coreness of a vertex is k if it belongs to the k-core but not to the (k+1)-core, a subgraph where every node has k connections.
Graph density	The density of a graph is the ratio of the number of edges and the number of possible edges.

## Availability and requirements

Source code and binaries freely available for download at http://cic.scu.edu.cn/bioinformatics/Pre-miRNA_code.zip

Operating systems: Platform independent

Programming language: Perl, R language

License: none

## Competing interests

The authors declare that they have no competing interests.

## Authors' contributions

ML initiated and guided the whole project. JX and XT developed the methods, wrote the source code, and drafted and revised the manuscript. YL, ZF, DM provided helpful insight in the method design and assessment of manuscript. YL and YH helped in the revision of the manuscript. All authors read and approved the final manuscript.
